# Application of Conducting Polymer Nanostructures to Electrochemical Biosensors

**DOI:** 10.3390/molecules25020307

**Published:** 2020-01-12

**Authors:** Waleed A. El-Said, Muhammad Abdelshakour, Jin-Ha Choi, Jeong-Woo Choi

**Affiliations:** 1 Department of Chemistry, Faculty of Science, Assiut University, Assiut 71516, Egypt; awaleedahmed@yahoo.com (W.A.E.-S.); muhammed.abdl_shakor@science.au.edu.eg (M.A.); 2 Department of Chemical and Biomolecular Engineering, Sogang University, 35 Baekbeom-Ro, Mapo-Gu, Seoul 04107, Korea; jinhachoi@sogang.ac.kr

**Keywords:** conducting polymers, molecularly imprinted polymer, cell-based chip, nanotechnology, electrochemical sensor, biosensors

## Abstract

Over the past few decades, nanostructured conducting polymers have received great attention in several application fields, including biosensors, microelectronics, polymer batteries, actuators, energy conversion, and biological applications due to their excellent conductivity, stability, and ease of preparation. In the bioengineering application field, the conducting polymers were reported as excellent matrixes for the functionalization of various biological molecules and thus enhanced their performances as biosensors. In addition, combinations of metals or metal oxides nanostructures with conducting polymers result in enhancing the stability and sensitivity as the biosensing platform. Therefore, several methods have been reported for developing homogeneous metal/metal oxide nanostructures thin layer on the conducting polymer surfaces. This review will introduce the fabrications of different conducting polymers nanostructures and their composites with different shapes. We will exhibit the different techniques that can be used to develop conducting polymers nanostructures and to investigate their chemical, physical and topographical effects. Among the various biosensors, we will focus on conducting polymer-integrated electrochemical biosensors for monitoring important biological targets such as DNA, proteins, peptides, and other biological biomarkers, in addition to their applications as cell-based chips. Furthermore, the fabrication and applications of the molecularly imprinted polymer-based biosensors will be addressed in this review.

## 1. Introduction

Conducting polymers (CPs) have emerged as one of the most promising materials in many biological and biomedical applications, including biosensors and tissue engineering applications [1,2,3]. The wide applications of the CPs are owing to their biocompatibility and their unique electrical properties that could convert the biochemical information into electrical signals. In addition, CPs have several functional groups, which provide maximum enzyme loading through the interaction between the enzyme molecules and the polymers’ functional groups, thus a well-organized scaffold biosensors could be achieved [4]. Recently, nanostructured CPs represented an excellent building block for developing highly sensitive biosensors [5] due to their unique properties that combine with those of the CPs (biocompatibility, direct electrochemical synthesis) and the nanomaterials (e.g., large surface area, flexibility for the immobilization of biomolecules and quantum effect) [6,7,8]. Several synthetic strategies were reported for the nanostructured CPs (NSCPs) synthesis, including template-based (either hard or soft template) methods, template-free synthesis [9,10,11,12], as well as the physical approaches (e.g., electrospinning) [13].

Among the many NSCPs, polyaniline nanostructures (PANI NSs) have been mainly prepared with the aid of template-guided polymerization within channels of microporous zeolites, porous membranes, and chemical route in the presence of self-organized supramolecules or stabilizers [14,15,16]. PANI NSs have higher sensitivity and faster time response than its conventional bulk counterpart due to higher active surface area and shorter penetration depth for target molecules [16]. The high surface area and porous structure further allow the fast diffusion of molecules into the framework leading to their applicability as biosensors. The blend of metal nanoparticles with conjugated polymers to form nanocomposite is intended to increase electrical conductivity [17,18]. One of the most significant current discussions has clearly demonstrated that gold and silver nanoparticles could be embedded into a polymeric layer, which largely increased the surface area for modification of diverse biomolecules. In recent years, there has been considerable interest in the system of electrode modification using nanoparticles and conducting polymers [18,19,20,21,22,23,24].

## 2. NSCP-Integrated Electrochemical Biosensors

### 2.1. Nscps for Electrochemical Detection of Glucose and H_2_O_2_

**Figure 1:**
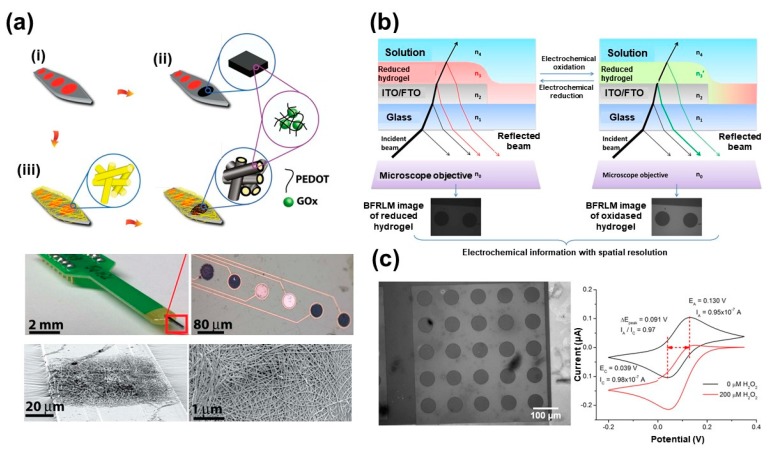
(**a**) Glucose oxidase (GOx)-incorporated PEDOT on the microelectrode array: (**i**) Pt microelectrode array. (**ii**) Electrodeposition of GOx-incorporated PEDOT film (PEDOT F-GOx) with electrospinning of PEDOT nanofibers (PEDOT NFs-GOx), poly(L-lactide) (PLLA) nanofibers on the microelectrode array. (**iii**) Electrodeposition of PEDOT around the PLLA nanofibers to form GOx-incorporated PEDOT nanofibers (PEDOT NF-GOx). Optical and scanning electron microscope (SEM) images of the entire microelectrode array are below Reproduced with permission [30]. Copyright 2014 Wiley. (**b**) Schematics of BFRLM for increasing the spatial resolution of redox hydrogel-based electrochemical biosensors. The incident light is refracted and reflected on the different interfaces of the multilayered sensor [32]. (**c**) Electrochemical detection of hydrogen peroxide using an Fluorine doped Tin Oxide (FTO) electrode modified with horseradish peroxidase (HRP)-based redox hydrogel [32].

Nowadays, diabetes mellitus represents a severe health problem worldwide due to its complications that are more harmful than diabetes itself [25]. Therefore, developing an accurate and fast assay for early diagnosis of diabetes disease is an urgently needed issue. Several analytical assays were reported for monitoring diabetes based on the measurement of glucose level in blood [26,27,28]. The glucose sensors could be classified into enzymatic and enzyme-free sensors [26,27,28,29]. Here, we will discuss the uses of CPs for developing highly sensitive glucose sensors. Deepshikha et al. report on the preparation of PANI NSs by using sodium dodecylsulphate (NSPANI-SDS) as glucose and H_2_O_2_ biosensor [15]. SDS acts as an ideal structure-directing agent for the synthesis of ordered nanostructured polymer composed of framework protonated amine such as PANI NSs. The uses of these NSs polymer with large specific surface area could enhance the conductivity of PANI and results in easily immobilization with high loading of horseradish peroxidase (HRP) and glucose oxidase (GOx). In addition, these NSs enhance the rate of electron transfer and the current response. These modified PANI NSs were used as optical and electrical biosensors with good performances, fast response time, wide linear range, and good selectivity, stability and reproducibility. Furthermore, Abidian et al. have reported the fabrication and applications of PEDOT nanofibers for electrochemical detection of glucose based on entrapped of the glucose oxidase enzyme (GOx) into the PEDOT nanofibers during the galvanostatic polymerization process at Pt electrode [30]. This sensor has demonstrated a high sensitivity, high electrochemical stability and lower limit of detection (LOD) than the GOx-incorporated PEDOT film (PEDOT F-GOx) sensors (as shown in Figure 1a) that related to their large surface area. Soganci et al. have modified the graphite rod electrode with a super-structured CP composed of amine substituted thienyl-pyrrole derivative based on the electropolymerization process [31]. This CP is characterized by the presence of free amine groups that allowed the covalent bonding between the electrode and the biorecognition elements such as glucose oxidase (GOx). This sensor was applied for glucose detection in beverages. Munteanu et al. showed a dual electrochemical sensor with optical microscopy as an opto-electrochemical sensor for detecting both hydrogen peroxide and glucose [32]. The principal of the sensor is based on uses of osmium complex-based redox polymer hence its oxidation state could change during the interactions of hydrogen peroxide and glucose with the enzyme, which is a time-dependant interaction as depicted in Figure 1b,c.

### 2.2. Nscps for Cell-Based Chip Applications

**Figure 2:**
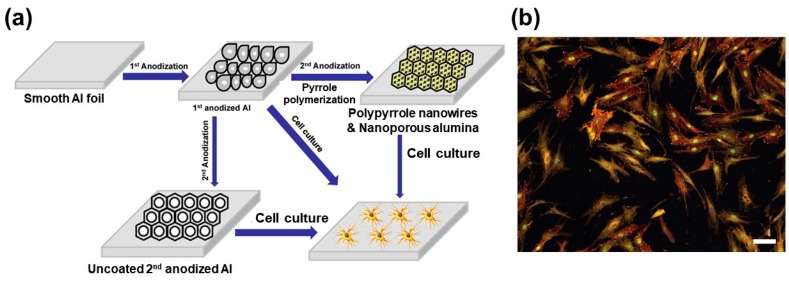
(**a**) Schematic diagram represented the fabrication of different cell culture substrates and the cell immobilization process. Reproduced with permission [37]. Copyright 2010 Elsevier. (**b**) Human fibroblasts after two days in culture on PPyThon film on gold. Green stains Ki67 (proliferation marker), yellow stains vinculin (focal adhesion marker), red stains actin (cytoskeleton). Nuclei counterstained with DAPI (blue). The scale bar is 100 µm. Reproduced with permission [38]. Copyright 2013 Elsevier.

It is challenging to understand cell behavior based on the measurement of only nucleic acid or protein expression levels because the cells are much more complicated than the sum of its components [33]. Several electrically conductive scaffolds have been used for making a cell-based chip for enhancing the adhesion, proliferation, and differentiation of several cell types such as neurons [33,34,35]. Here, we will address the uses of CPs modified electrodes for developing cell-based chips and their applications. Lee et al. developed an electrochemical conducting scaffold composed of pyrrole N-hydroxyl succinimidyl ester and pyrrole (PPy-NSE) copolymer, then modified this copolymer with nerve growth factor (NGF) and it used for PC12 cells immobilizations [36]. They have claimed that cells have extended neurites similarly to that for cells cultured in medium containing NGF. El-Said et al. reported on uses of a thin layer of polyaniline emeraldine base (EB) coated indium–tin oxide (ITO) electrode as a cell-based chip [35]. On the contrary to the metal electrodes, PANI-EB/ITO electrode showed an excellent electrochemical activity at neutral pH without co-deposition of an acidic counterion. The developed electrode was used as a cell-based chip for quick and easy measuring cellular electrochemical properties, the cell viability, cell adhesion, cell proliferation and monitoring the effects of different anticancer drugs on the cell viability. The same group has reported the in-situ electrochemical synthesis of polypyrrole (PPy) nanowires with a nanoporous alumina template [37]. They have shown the formation of highly ordered porous alumina substrate and the growth of the PPy nanowires inside the nanoporous structures based on the direct electrochemical oxidation of the pyrrole monomer, as shown in Figure 2a. The cellular behavior, cell morphology, adhesion, and proliferation, as well as the biocompatibility of PPy nanowires/nanoporous alumina substrate towards both cancerous and normal cells, were investigated compared with other substrates. They have demonstrated that the PPy nanowires/ nanoporous alumina substrate showed better cell adhesion and proliferation than other control substrates. This study showed the potential of the PPy nanowires/nanoporous alumina substrate as biocompatibility electroactive polymer substrate for both healthy and cancer cell cultures applications. Strover et al. have incorporated pyrrole and thiophene moieties in its monomer, to fabricated PolyPyThon (PPyThon)-based molecular brushes [38]. A film of this CP was deposited on the gold substrate used as a scaffold for electrical stimuli-responsive surfaces of human fibroblasts cells, as shown in Figure 2b.

Based on the above, uses of the cell-based chips is a promising alternative to animal experiments, due to the disadvantages of the uses of animal models because they are violating animals’ rights, costly, time-consuming and also poor relevance to human biology. In addition, the uses of cell-based chips as biosensors for monitoring effects of anticancer drugs or for monitoring the differentiation of the neural cells showed many advantages that including (1) increasingly more sophisticated representation of absorption, distribution, metabolism, excretion, and toxicity (ADMET) process, (2) better understand the drug interaction mechanisms in the human body, and (3) showed a great potential to better predict drug efficacy and safety.

### 2.3. Nscps for Different Biosensor Applications

**Figure 3:**
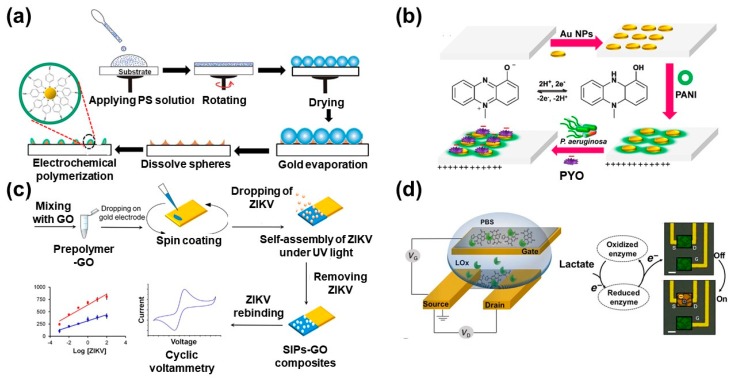
(**a**) Preparation of PATP nanostructures patterns gold modified Indium Tin Oxide. Reproduced with permission [39]. Copyright 2014 Elsevier. (**b**) The schematic diagram for the fabrication of polyaniline (PANI)/Au NPs/ITO electrode and the interaction between PANI and pyocyanin (Pyocyanin (PYO) [43]. (**c**) Scheme depicting surface imprinted polymers (SIPs)-graphene oxide composites preparation on a gold surface for making the ZIKV biosensor. Reproduced with permission [44]. Copyright 2019 ACS publication. (**d**) Schematic of the organic electrochemical transistor (OECT) (gate dimensions, 500 mm^2^; channel dimensions, 10 mm (length) × 100 mm (width) × ~100 nm (thickness)) [45].

Conducting polymers have been widely used for preparing of sensor platforms and imparts many advantages due to the incorporation of their functional groups into their fabrication. Here, we will discuss the fabrication and uses of different NSCPs and their composites, as well as their electrochemical biosensor applications for the various biological targets such as nucleic acid, ATP, neurotransmitter, etc. Guanine (G) and adenine (A) and are two of the purine bases, which participate in the building of nucleic acids and are fundamental compounds in different biological systems. The abnormal concentration of A and G in body fluid is related to the deficiency of the immunity system. Hence, monitoring of the A and G concentration in living organisms is a great demand issue. El-Said et al. have fabricated poly(4–aminothiophenol) (PATP) nanostructures layered on gold nanodots patterned indium tin oxide (ITO) electrode based on a simple method as shown in Figure 3a [39]. The modified gold nanodots ITO electrode was fabricated based on thermal evaporation of pure Au metal onto the ITO surface through polystyrene monolayer. Then, use of these Au nanodots as a template for self–assembly immobilization of ATP molecules followed by electrochemical polymerization of ATP into PATP. The modified electrode was applied to monitor the concentration of the mixture of adenine and guanine with LOD of 500 and 250 nM, respectively. Furthermore, the modified electrode was extensively applied for detecting adenine and guanine in human serum.

Aksoy et al. have developed a selective electrochemical dopamine biosensor based on polyimide (PI) and polyimide-boron nitride (PI-BN) composites as a selective membrane for dopamine detection [40]. The introduction of BN particles into the PI matrix results in enhancement of the sensitivity, selectivity, and reversibility (i.e., the rapid electron transfer), with a LOD of about 4 × 10^−8^ M. Hybridization of PANI with nanomaterials could endow great promise in the sensors field due to the enhancement of its electrical conductivity in addition to its capability to act as a scaffold for immobilization of the biological species [41]. *Pseudomonas aeruginosa* (*P. aeruginosa*) is among the most common pathogenic gram-negative bacteria that could cause corneal ulcers and blindness within two days. Pyocyanin (PYO) is the biomarker that has been used for monitoring *P. aeruginosa*. Elkhawaga et al. have prepared PANI/Au NPs/ITO electrode as PYO sensor in a culture of *P. aeruginosa*. The results indicated that PANI/Au NPs/ ITO electrode is more sensitive toward PYO biomarker than either bare ITO electrode or Au NPs/ITO electrode with LOD of 500 nM [42]. The enhancement of the electrochemical activity of PANI/Au NPs/ITO modified electrode towards the PYO related to the presence of positive charges on its surface that could enhance the mass transfer rate of the negatively charged PYO based on the electrostatic attraction force. The same group has extended the uses of PANI/Au NPs/ ITO electrode for diagnosis of *P. aeruginosa* in 50 samples collected from patients suffering from corneal ulcers as shown in Figure 3b [43]. The obtained results were compared with the results gained by the screen-printed electrode (SPE), conventional techniques, automated identification method, and the amplification of the 16 s rRNA gene by polymerase chain reaction (PCR) as a standard test for *P. aeruginosa* identification. The electrochemical detection of PYO by square wave voltammetry (SWV) technique using PANI/Au NPs modified ITO electrode was the only technique showing 100% agreement with the molecular method in sensitivity, specificity, positive and negative predictive values when compared with the SPE, conventional (including colony morphology, pigment production, and biochemical tests) and automated (including the automated ID and Ast system and the PCR) methods. Thus, PANI/Au NPs/ITO electrode is recommended as a fast, cheap, accurate, and selective PYO biomarker sensor in *P. aeruginosa* in the corneal ulcer cases based on the SWV technique.

Zika virus (ZIKV) is a flavivirus. Recently, there is an increasing interest in developing a rapid Zika virus identification assay due to the appearance of the viral infection in infants. Tancharoen et al. report the development of a new type of ZIKV electrochemical biosensor [44]. This biosensor consisted of surface imprinted polymers (SIPs)/graphene oxide composites, as shown in Figure 3c. The LOD of this sensor agreed with that of the RT-PCR method, in addition to the capability of this sensor to detect the Zika virus in the presence of the dengue virus and serum samples.

Lactate is one of the cellular metabolites, which is associated with some critical health care conditions. Pappa et al. reported the fabrication of a micrometer-scale polymer-based transistor platform for the detection of lactate, as shown in Figure 3d [45]. The uses of the electron-transporting (n-type) organic polymer incorporate hydrophilic side chains that have enhanced the ion transport/injection, facilitated the enzyme conjugation and acted as a series of redox centers. The developed sensor showed a fast, selective, and sensitive metabolite capability.

## 3. Molecularly Imprinted Polymers as Selective Biosensors

**Figure 4:**
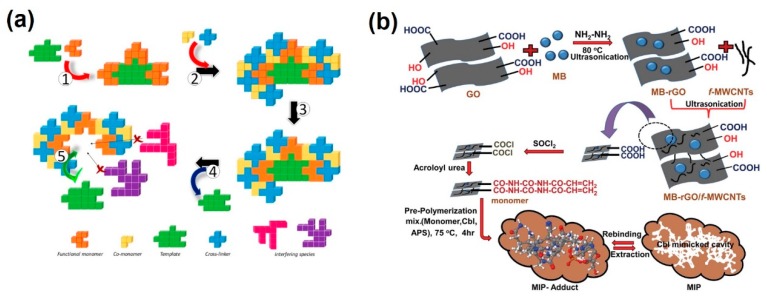
(**a**) Scheme for the preparation of a molecularly imprinted polymer (MIP). (**1**) First, template molecule (T) and functional monomers (FM) are mixed to get the complex FM-T. (**2**) The rest of the components of the pre-polymerization mixture are added, i.e., co-monomers (CM) and cross-linker (CL). (**3**) After the addition of a radical initiator (thermal or photochemical) or a catalyst (acid or base), polymerization takes place. (**4**) T is removed resulting in a pocket within the polymeric matrix. (**5**) T re-binds the selective cavity while other potential interfering species are blocked due to limitations in terms of size, shape and/or functional group distribution [46]. (**b**) Schematic presentation of MB-rGO/f-MWCNTs/AU-MIP Development. Reproduced with permission [52]. Copyright 2018 ACS publication.

Molecularly imprinted polymers (MIPs) were reported as one of the most sensitive and selective materials for biosensor applications [46]. The fabrication process of the MIPs involved the uses of three components (i) the template molecules (imprinted molecules), (ii) the functional monomers (FM) and (iii) the cross-linker molecules. The fabrication process of the MIPs and its recognition uses was summarized in Figure 4a, in which the template molecules (imprinted molecules) combined with the functional monomer to form multiple reaction sites. The polymerization process was performed in the presence of a cross-linker. The template molecules were removed from the resultant polymers to form imprinted cavities that could rebind with the template molecules during the recognition process. The selectivity mechanism of the MIPs is based on the fact that these MIPs offered structurally adapted recognition sites for the rebinding of target molecules such as drugs in complex mixtures. The selectivity of the MIPs to molecules is related to their matching in chemical groups and the interactions between template molecules and imprinted groups that depend on the shape and rigidity of the template molecules. Therefore, these cavities could be used for enantiomeric resolution (i.e., differentiate between the S-type and R-type analogs (optical enantiomers)).

MIPs were used for electrochemical recognition of different targets [47,48,49]. Cyanocobalamin (Cbl) is one of the eight B-complex vitamins that could affect the brain function and the entire nervous system. The decrease of the Cbl levels reflects several health problems such as hematological and neurological disorders [50,51]. Singh et al. have reported on fabrication of a biomimetic imprinted polymer consisted of a sheet of reduced graphene oxide (rGO) decorated with multiwalled carbon nanotubes and methyl blue composite and functionalized with poly acryloylurea as shown in Figure 4b [52]. This sensor was applied for ultra-trace detection of Cbl based on differential-pulse voltammetry (DPV) technique, which demonstrated a linearity relationship within a concentration range from 0.021 to 1.44 ng/mL with LOD of 0.0056 ng/mL and absolutely no interference in real samples. The high electrochemical conductivity of this sensor related to the interaction between methyl blue/rGO could and the f-MWCNTs based on π stacking, and the high solubility of the MB-rGO/f-MWCNTs composite in water due to the presence of the hydrophilic sulphonate (SO^3−^ groups).

Nonylphenol is one of the phenolic environmental estrogens, which could affect different organisms’ systems, including the endocrine systems, the immune and reproductive system [53,54,55]. Liu et al. reported on the fabrication of MIP-modified glassy carbon electrode as an electrochemical nonylphenol sensor [56]. The MIP electrode consisted of the surface of acrylamide-functionalized multi-walled carbon nanotubes (MWNT) as a carrier surface, nonylphenol as a template molecule, methacrylic acid as functional monomer and ethylene glycol dimethacrylate as a crosslinking agent and the polymerization was performed based on the thermal polymerization process. This sensor showed a linear range within the concentration range from 0.1 to 30 μM with a LOD of 0.02 μM.

## 4. Conclusions

Here we have discussed the synthesis and uses of conducting polymers nanostructured as well as their biosensors and biological applications. Uses of the conducting polymers nanostructures in the biosensors field related to their excellent conductivity, stability, and ease of preparation. One of the important inherent specificity of enzyme-based electrochemical biosensors is that the uses of biorecognition elements are to choose suitable matrixes for immobilization of the biorecognition elements. The conducting polymers could provide excellent matrixes for immobilization of different biorecognition elements molecules (e.g., enzymes) with keeping their redox centers in excellent electrical communication with the transducing electrode and hence enhance their biosensors efficiency. Furthermore, combinations of metals nanostructures with conducting polymers result in enhancing the stability of the nanostructures composites. Here we have shown the synthesis and application of several metals/conducting polymer nanostructures. Especially the electrochemical biosensor applications of the conducting polymers nanostructures for monitoring different important biological target species such as DNA bases, proteins, peptides, glucose, viruses, and cell-based chips. Moreover, we have discussed the fabrication of the molecularly imprinted polymer-based biosensors.
